# Magnetic Properties of Gd-Doped Bi_7_Fe_3_Ti_3_O_21_ Aurivillius-Type Ceramics

**DOI:** 10.3390/ma17153760

**Published:** 2024-07-30

**Authors:** Joanna A. Bartkowska, Diana Szalbot, Jolanta Makowska, Małgorzata Adamczyk-Habrajska, Zbigniew Stokłosa

**Affiliations:** Institute of Materials Engineering, Faculty of Science and Technology, University of Silesia, 75 Pułku Piechoty 1A, 41-500 Chorzow, Poland; joanna.bartkowska@us.edu.pl (J.A.B.); diana.szalbot@gmail.com (D.S.); malgorzata.adamczyk-habrajska@us.edu.pl (M.A.-H.); zbigniew.stoklosa@us.edu.pl (Z.S.)

**Keywords:** Aurivillius phases, magnetic properties, hysteresis loop

## Abstract

The magnetic properties of Aurivillius-phase Bi_7_Fe_3_Ti3O_21_ (BFT) and Bi_7−x_Gd_x_Fe3Ti3O_21_, where *x* = 0.2, 0.4, and 0.6 (BGFT), were investigated. Ceramic material undoped (BGF) and doped with Gd^3+^ ions were prepared by conventional solid-state reaction. In order to confirm that the obtained materials belong to Aurivillius structures, XRD tests were performed. The XRD results confirmed that both the undoped and the gadolinium-doped materials belong to the Aurivillius phases. The qualitative chemical composition of the obtained materials was confirmed based on EDS tests. The temperature dependences of magnetization and magnetic susceptibility were examined for the ceramic material both undoped and doped with Gd^3+^ ions. The measurements were taken in the temperature range from *T* = 10 K to *T* = 300 K. Using Curie’s law, the value of the Curie constant was determined, and on its basis, the number of iron ions that take part in magnetic processes was calculated. The value of Curie constant C = 0.266 K, while the concentration of iron ions Fe^3+^, which influence the magnetic properties of the material, is equal 3.7 mol% (for BFT). Hysteresis loop measurements were also performed at temperatures of *T* = 10 K, *T* = 77 K, and *T* = 300 K. The dependence of magnetization on the magnetic field was described by the Brillouin function, and on its basis, the concentration of Fe^3+^ ions, which are involved in magnetic properties, was also calculated (3.4 mol% for BFT). Tests showed that the material is characterized by magnetic properties at low temperatures. At room temperature (RT), it has paramagnetic properties. It was also found that Gd^3+^ ions improve the magnetic properties of tested material.

## 1. Introduction

Aurivillius-type materials are layered perovskite-like crystalline structures [1], which can be described by the general formula BiO-O [1]. This type of structure is characterized by the formation of octahedral connections (O) between bismuth-oxide (BiO) layers, which are similar to the structure of perovskite [2,3]. Bismuth perovskite-like layers, called Aurivillius phases, have become the subject of great interest in recent years due to the potential possibility of having both electrical and magnetic properties in one phase [4,5,6,7,8,9,10]. Due to these properties, magnetoelectric Aurivillius-type structures can be classified as multiferroic materials [11,12,13]. In turn, multiferroics are a very promising group of materials for applications in modern electronics and spintronics [14]. Multiferroic Aurivillius-type materials with magnetic and electric properties may have potential applications in new types of switches, sensors, memory storage devices, and random access memory (FeRAM) [15,16,17,18,19,20,21,22]. An interesting material with an Aurivillius-type structure is a four-layer material doped with gadolinium: Bi_4_Gd_2_Ti_3_Fe_2_O_18_ [23]. The authors obtained a perovskite-like four-layer structure of the Aurivillius type that was doped with gadolinium. In the presented research, the authors showed that the obtained material is characterized by magnetic properties (paramagnetic and weak ferromagnetic). By appropriately selecting technological conditions and the amount of dopant ions, the physical properties of the material can be influenced.

The magnetic research presented in this paper concerns synthesized samples of the Aurivillius-type ceramic material with the chemical formula Bi_7_Fe_3_Ti_3_O_21_, for which results regarding phase analysis, microstructure, and electrical properties have already been published in the paper [24].

The authors showed that doping this material with Gd^3+^ ions increases the value of the electric constant. The research presented in [24] indicated that the ceramic material with an Aurivillius-type structure is characterized by electrical properties and weak electromagnetic coupling, which also indicates the presence of magnetic properties.

The research subject presented in this paper was the magnetic properties of seven-layer Aurivillius-type structures doped with gadolinium ions (Gd^3+^). The work aimed to obtain Aurivillius-type ceramics doped with gadolinium ions (Gd^3+^) and to investigate its magnetic properties, such as magnetization both as a function of temperature and as a function of the magnetic field, and magnetic susceptibility.

Improving magnetic properties by doping the base material with Gd^3+^ ions may have great application significance because, as shown in [24], doping BFT with Gd^3+^ ions improved the electrical properties of the ceramic material. The authors tried to obtain a material that would be classified as multiferroic material; i.e., in addition to good electrical properties, it would also have good magnetic properties.

The work also aimed to determine what mole % of Fe^3+^ ions is involved in magnetic processes and what effect Gd^3+^ ions have on the magnetic properties. This paper presents the conditions of the technological process for obtaining Aurivillius-type ceramic material and the results of the research on the influence of doping a seven-layer Aurivillius-type structure with gadolinium ions (Gd^3+^) on its microstructure and magnetic properties.

## 2. Materials and Methods

All Aurivillius-type ceramic materials discussed in this article, namely Bi_7_Fe_3_Ti_3_O_21_ (BFT) and Bi_7−x_Gd_x_Fe_3_Ti_3_O_21_, where *x* = 0.2, 0.4, and 0.6 (BGFT2, BGFT4, and BGFT6, respectively), were synthesized by solid-state synthesis reactions from a mixture of simple oxides, and the produced ceramic powders were densified by free sintering. The starting substrates for the production of undoped and doped Bi_7−x_Gd_x_Fe_3_Ti_3_O_21_ (*x* = 0, 0.2, 0.4, and 0.6) ceramics were the following oxides: Fe_2_O_3_ (Sigma-Aldrich, St. Louis, MO, USA), purity 99%), TiO_2_ (POCH, Gliwice, Poland, purity 99.9%), Bi_2_O_3_ (Aldrich, St. Louis, MO, USA, purity 99.9%), and Gd_2_O_3_ (Aldrich Chemistry, St. Louis, MO, USA, purity 99.9%).

The substrates were weighed in a stoichiometric ratio using an electronic analytical balance RADWAG PS750/X (Radom, Poland). The weighed oxides were pre-mixed in a porcelain mortar for *t* = 45 min. The obtained mixtures were put into polyamide cups with zirconium–yttrium grinders (balls with a diameter of *ϕ* = 10 mm), and ethyl alcohol C_2_H_5_OH (POCH, Gliwice, Poland, purity 99.99%,) was added. The cups and their contents were placed in a planetary ball mill. The substrate mixture was wet-milled for *t* = 24 h. The operating speed of the device was *υ* = 250 rpm. The role of the long grinding time was to create a homogeneous mixture. After wet mixing, the powder was dried in air for *t* = 24 h and remixed in a porcelain mortar for *t* = 30 min. The details of the technological process have already been presented in detail in previous works [25,26].

The microstructure analysis of all obtained materials was performed using a scanning electron microscope (JEOL JSM-7100F TTL LV, Jeol Ltd., Tokyo, Japan). This microscope was equipped with an energy-dispersive spectrometer (EDS). Qualitative and quantitative assessment of the chemical composition was carried out using X-ray analysis. Magnetic properties were obtained by applying the Quantum Design PPMS system (Quantum Design, PPMS 7T ACMS module, San Diego, CA, USA). The magnetization was measured in the temperature range from *T* = 10 K to *T* = 300 K and in an external magnetic field of *H* = 10 kOe. The magnetic hysteresis loops were measured at temperatures of *T* = 10 K, *T* = 77 K, and *T* = 300 K and in the magnetic field *H* range between *H*= −20 kOe and *H* = +20 kOe.

## 3. Results and Discussion

### 3.1. Structure and Microstructure Studies

X-ray studies showed that a single-phase ceramic was obtained, characterized by an orthorhombic crystal structure for all compounds with the *Fm*2*m* space group. The unit cell parameters are described in the paper [24].

**Figure 1:**
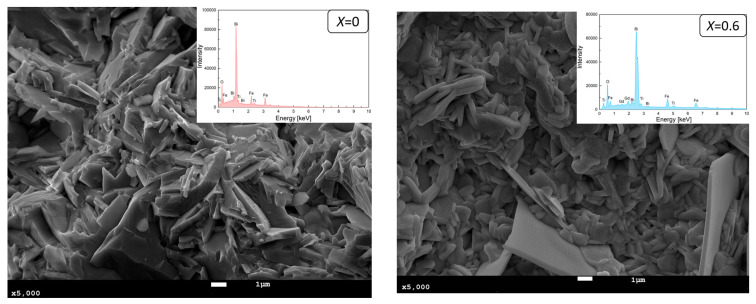
EDS spectrum and SEM photographs of fracture of BFT and BGFT6 ceramic materials.

Figure 1 shows EDS spectrum and SEM photographs of the fracture of BFT and gadolinium-doped ceramics with a concentration of *x* = 0.6 at 5000× magnification. As noted previously [25,26], BFT ceramics exhibit plate-like grains typical of Aurivillius phases, with closely packed plates contributing to grain fracture paths, indicating well-sintered and robust grains with defined boundaries. Doping with gadolinium (Gd^3+^) significantly alters the microstructure, transforming plate shapes to rounded forms and creating a more chaotic grain distribution. Additionally, grain size becomes more heterogeneous [24].

EDS analysis verified the qualitative and quantitative chemical composition of the synthesized Bi_7−x_Gd_x_Ti_3_O_21_ ceramics, confirming the absence of foreign elements or impurities. Therefore, it can be concluded that the Bi_7−x_Gd_x_Ti_3_O_21_ ceramics preserve the intended chemical composition. The applied technology enabled the production of chemically homogeneous materials that adhere to the specified stoichiometry [24].

### 3.2. Magnetic Properties

**Figure 2:**
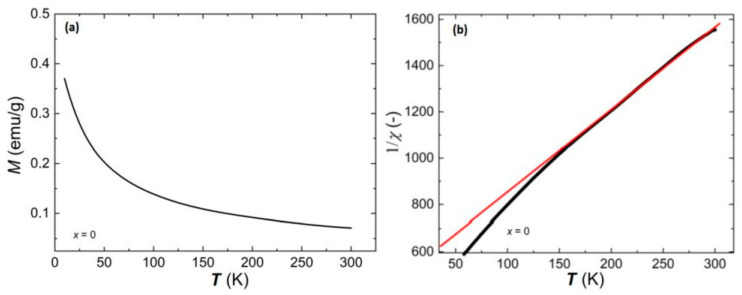
(**a**) Temperature dependence of magnetization for Bi_7_Fe_3_Ti_3_O_21_ ceramics, an external magnetic field with intensity *H* = 10 kOe; (**b**) temperature dependence of the inverse of magnetic susceptibility for Bi_7_Fe_3_Ti_3_O_21_ ceramics. Red line represents data obtained after fitting of Curie’s law.

The study of the magnetic properties of Bi_7_Fe_3_Ti_3_O_21_ ceramics began with measurements of magnetization as a function of temperature in the cooling process starting from room temperature (RT) up to *T* = 10 K and inverse of magnetic susceptibility as a function of temperature in a measurement field with an intensity of *H* = 10 kOe. The obtained results are presented in Figure 2.

The course of the curve presented in Figure 2a is characteristic of paramagnetic materials [27]. Figure 2b shows that in the temperature range from *T* = 150 K to *T* = 300 K, the relationship in question is linear, while below the temperature *T* = 150 K, a significant departure from linearity is noticeable. A similar shape of the relationship *χ*^−1^(*T*) was presented by the authors of [28]. The linear nature of the relationship in the higher temperature range allows us to conclude that it meets Curie’s law. Approximation of the obtained characteristics with a linear function allowed us to determine the value of the Curie constant, which is *C* = 0.266 K.

Magnetization can be expressed by the following relationship (1) [29]:

(1) $$M≅\frac{\mu_{o}N\mu^{2}H}{3k_{B}T}=\frac{C}{T},$$

where *μ_o_* is a vacuum magnetic permeability, *N* is number of atoms per unit volume, *μ* is a magnetic moment of the atom, *H* is a magnetic field, *k*_B_ means the Boltzmann constant, *T* is the temperature, and *C* is the Curie constant.

Using the relationship (Equation (1)) and knowing the value of Curie’s constant, the mole % of magnetic ions that influence the magnetic properties of the tested material was determined. Based on the approximation, it was calculated that (3.7 ± 0.2) mol% of all iron Fe^3+^ ions are involved in the magnetic processes occurring in the tested material. Additionally, by analyzing the course of the line fitting the relationship *χ*^−1^(*T*) to the experimental data in the temperature range *T* = (150–300) K, it can be seen that its extrapolation towards lower temperatures gives a negative value of intersection with the temperature axis. This behavior indicates the antiferromagnetic nature of the material [28,30,31].

**Figure 3:**
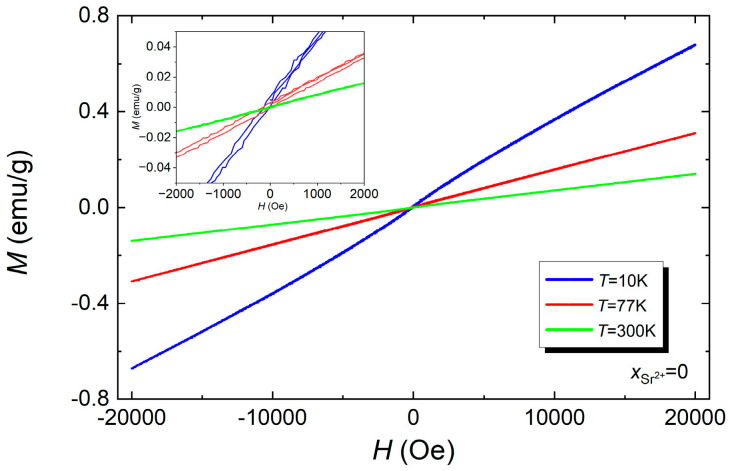
Dependencies of magnetization as a function of the applied magnetic field of BFT ceramics at temperatures *T* = 10 K, *T* = 77 K, and *T* = 300 K.

The magnetization *M* was measured as a function of the applied magnetic field with an intensity *H* ranging from *H* = −20 kOe to *H* = +20 kOe. Measurements of hysteresis loops were made at three selected temperatures T, namely *T* = 10 K, *T* = 77 K, and *T* = 300 K. The test results are presented in Figure 3.

The analysis presented in Figure 3 of the characteristics reveals that at temperature *T* = 300 K, BFT ceramics practically do not have hysteresis loops, which confirms their paramagnetic nature [32]. At temperatures lower than room temperature, the measurement points are arranged in narrow hysteresis loops, which indicates the presence of weak magnetic order in the tested material.

**Figure 4:**
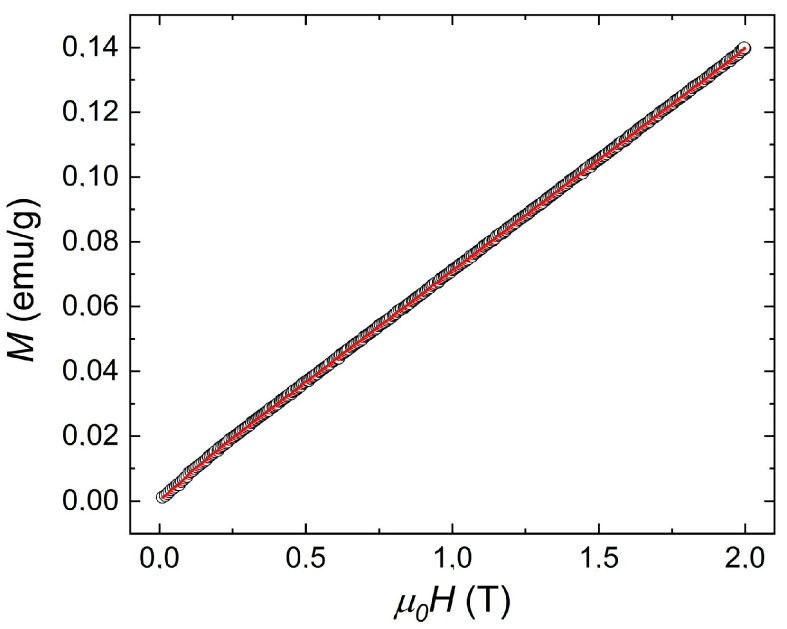
Dependence of primary magnetization on the applied external magnetic field at temperature *T* = 300 K (solid red line indicates fitting line).

To verify the amount of Fe^3+^ magnetic ions that contribute to the appearance of magnetic properties, calculations of their amount (mol%) were made using the dependence of the primary magnetization on the applied magnetic field (Figure 4).

The dependence of the magnetization on the external magnetic field can be expressed using the Brillouin’s function. This relationship has the following form (2) and (3) [29]:

(2) $$M=NgJ\mu_{B}\left(\frac{2J+1}{2J}\coth\left(\frac{2J+1}{2J}y\right)-\frac{1}{2J}\coth\left(\frac{y}{2J}\right)\right),$$

(3) $$\mathrm{with}\;y=\frac{gJ\mu_{B}H}{k_{B}T}$$

In these equations (Equation (2)), *N* is the number of atoms per unit volume, *g* is the Landé factor, *μ_B_* is the Bohr magneton, *J* is the total angular momentum of the magnetic ion, and *k_B_* is the Boltzmann constant.

By fitting the above equation (Equation (2)) to the measurement data of the primary magnetization as a function of the magnetic field (Figure 4), it is also possible to calculate the number of magnetic ions Fe^3+^ that participate in the magnetic properties. The number of iron ions that take part in magnetic phenomena determined based on the above fitting is (3.4 ± 0.1) mol%. Accurate to the measurement uncertainty, the number of Fe^3+^ ions (mol %) that take part in the magnetic properties, as calculated by both methods (namely using Curie’s law fitting and Brillouin’s function fitting), is consistent.

**Figure 5:**
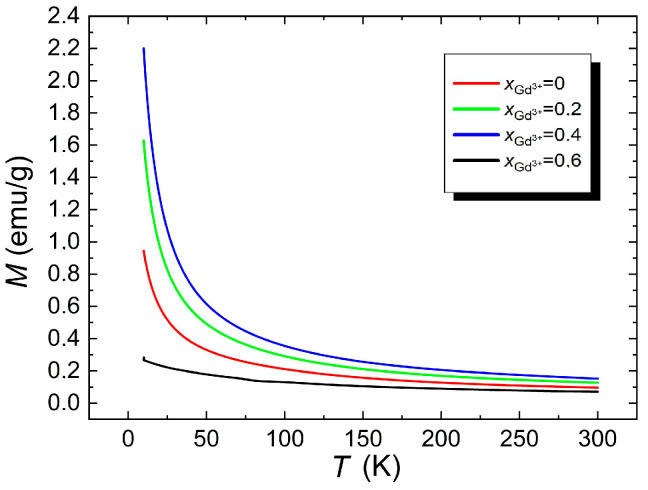
Temperature dependences of magnetization measured in a constant magnetic field *H* = 10^4^ Oe for Bi_7−x_Gd_x_Ti_3_O_21_, where *x*= (0, 0.2, 0.4, and 0.6).

In order to determine the effect of doping the BFT material with gadolinium ions Gd^3+^ on its magnetic properties, magnetization measurements were performed as a function of temperature. The measurement results are shown in Figure 5.

Figure 5 shows that with increasing Gd^3+^ dopant ions, the magnetization character changes from paramagnetic (for *x* = 0), showing possible antiferromagnetic ordering at low temperatures, to ferromagnetic for all amounts of gadolinium dopants. As the amount of dopant ions increases, an increase in the magnetization value *M* is visible.

Doping the ceramics BFT with magnetically active Gd^3+^ ions, which were incorporated in place of bismuth, improved the magnetic properties of the BGFT materials. The reason for this could be the fact that the ionic radius of gadolinium is smaller than the ionic radius of Bi^3+^, and coupling between Gd^3+^ and Fe^3+^ could also occur.

**Figure 6:**
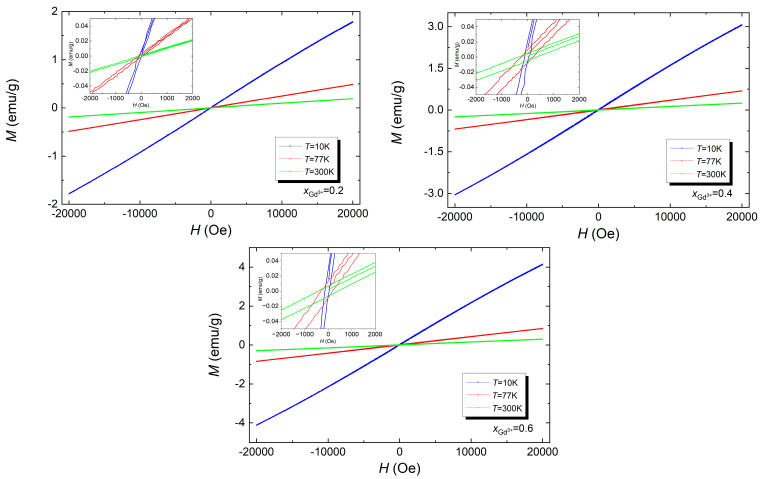
Dependence of the magnetization *M* on the applied magnetic field for Bi_7−x_Gd_x_Fe_3_Ti_3_O_21_, where *x* = 0.2, 0.4, and 0.6.

To further analyze the magnetic properties of the BFT material and the influence of the dopant on the magnetic properties (BGFT), the course of magnetization as a function of the magnetic field intensity was examined, i.e., hysteresis loops at temperatures *T* = 10 K, *T* = 77 K, and *T* = 300 K in the measurement magnetic field *H* range from *H* = −20 kOe to *H* = + 20 kOe. The results of these measurements are presented in Figure 6.

Figure 6 shows that the shape of the hysteresis loop for most materials shows an almost linear relationship, which supports the antiferromagnetic nature of these materials [33]. As the temperature increases, the width of the hysteresis loop increases for all materials doped with gadolinium ions. The widest hysteresis loops are observed at temperature *T* = 77 K. Based on the measurements presented in Figure 6, it can be concluded that doping BFT ceramics with Gd^3+^ ions improves the magnetic properties. The widest hysteresis loop shows BFT4 and BFT6 at room temperature *T* = 300 K, which indicates the typically ferromagnetic nature of these materials.

**Figure 7:**
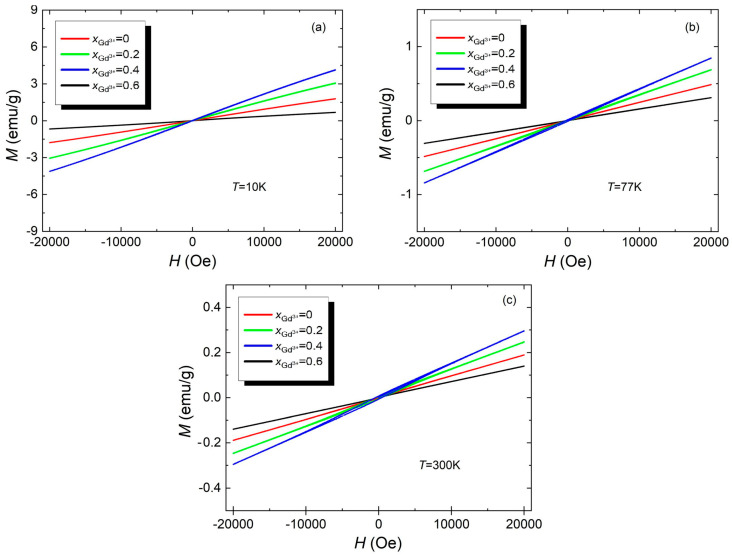
Hysteresis loops for all tested materials, i.e., BFT, BFT2, BFT4, and BFT6, measured at temperatures *T* = 10 K (**a**), *T* = 77 K (**b**), and *T* = 300K (**c**).

To show what effect the admixture of Gd^3+^ ions has on the magnetic properties of the tested materials, a juxtaposition of the hysteresis loop measurement results was made for undoped material and all amounts of dopant at selected temperatures, i.e., *T* = 10 K, *T* = 77 K, and *T* = 300 K. This juxtaposition is shown in Figure 7.

Figure 7 shows that the addition of gadolinium ions Gd^3+^ improved the magnetic properties of the tested materials at all temperatures. The reason for the improved magnetic properties may be the structural distortion caused by the substitution of gadolinium ions Gd^3+^ in place of bismuth ions Bi^3+^ because this causes a change in the Fe^3+^–O_2_–Fe^3+^ bond angle [34,35]. This, in turn, leads to the limitation of the spatially modulated, spiral spin structure, which results in weak ferromagnetism, as in the case of the Bi_1−x_Gd_x_FeO_3_ material [34]. The increase in the magnetization value with the increase in the amount of Gd^3+^ ions added may also be caused by the occurrence of antiparallel spin clusters. These spins can rotate in the direction of the magnetic field, resulting in ferromagnetic order [36]. The improvement in magnetic properties may also be caused by the existence of coupling between coexisting phases that occur around the structural transition boundary. This phenomenon is analogous to that occurring on the morphotropic phase boundary (MPB) [37].

**Figure 8:**
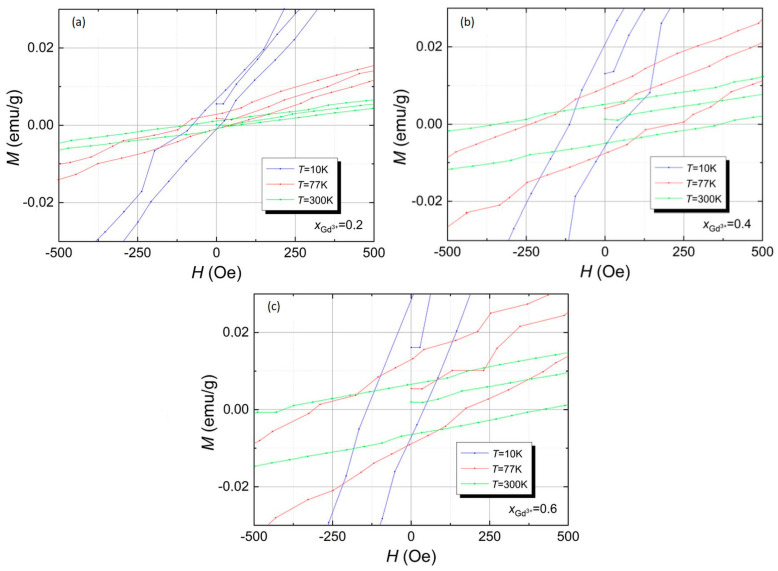
Hysteresis loops for all tested materials, i.e., BFT, BFT2, BFT4, and BFT6, measured at temperatures 10 K (**a**), 77 K (**b**), and 300 K (**c**) in the area of weak magnetic fields.

A detailed analysis of the hysteresis loops in the area of weak magnetic fields revealed that the hysteresis loops are not symmetrical. They are shifted towards the magnetic field axis, as shown in Figure 8.

This proves that the tested materials exhibit the effect of one-way exchange anisotropy, i.e., the so-called exchange bias [38,39]. The values of the basic quantities characterizing hysteresis loops for all gadolinium-doped materials are summarized in Table 1.

**Figure 9:**
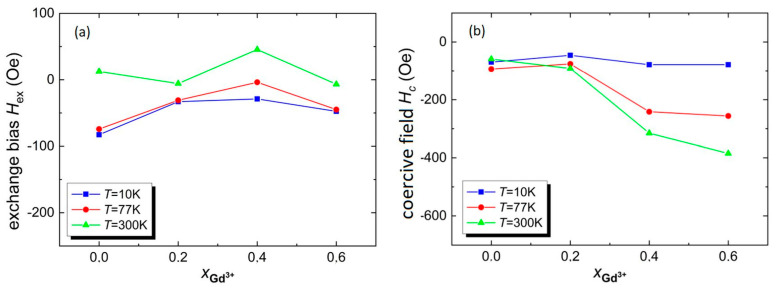
(**a**) Dependence of the field *H_ex_* for materials on the amount of admixture Gd^3+^ for BFT, BGFT2, BGFT4, and BGFT6; (**b**) dependence of the coercive field *H_c_* for materials on the amount of admixture Gd^3+^ for BFT, BGFT2, BGFT4, and BGFT6 at temperatures *T* = 10 K, *T* = 77 K, and *T* = 300 K.

The influence of the amount of Gd^3+^ ions on the values of the exchange bias field *H_ex_* and coercive field *H_c_* is shown in Figure 9a,b respectively.

Figure 9 shows that doping BFT ceramics with gadolinium ions Gd^3+^ causes both a negative field of exchange bias (*H_ex_* < 0) and a positive field exchange bias (*H_ex_* > 0). At temperatures *T* = 10 K and *T* = 77 K, the absolute value of the one-way exchange anisotropy field first decreases (for the dopant *x* = 0.2 and *x* = 0.4) and then increases for *x* = 0.6. However, at temperature *T* = 300 K, in the case of the BGFT2 material, the absolute value of *H_ex_* decreases, and then for BGFT4, the value of *H_ex_* increases rapidly and then decreases again for BGFT6. Ceramic material BGFT6 tested at temperature *T* = 10 K is characterized by the largest shift of the hysteresis loop in the direction opposite to the applied field, namely *H_ex_* =−47.50 Oe. The same ceramic material, however, for the temperature *T* = 300 K, shows the largest shift of the hysteresis loop in the positive direction, i.e., *H_ex_* = 45.55 Oe.

**Figure 10:**
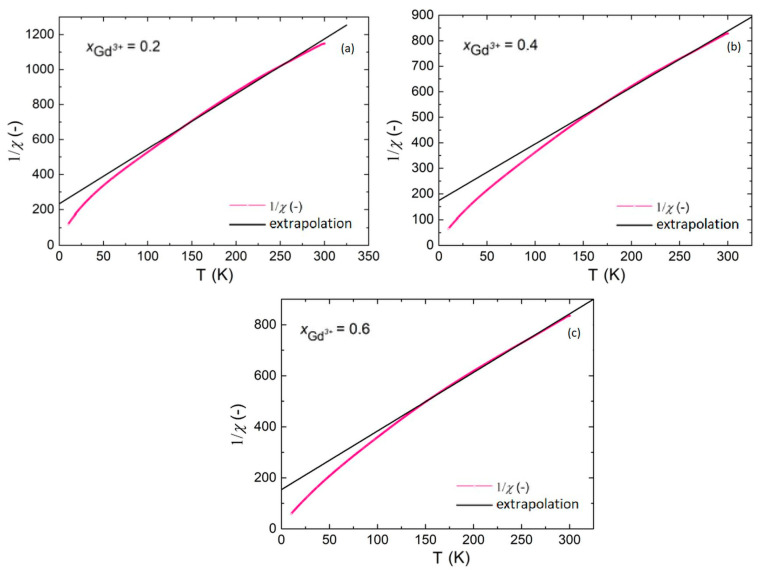
Dependence of the inverse of magnetic susceptibility on temperature for BGFT2, BGFT4, and BGFT6 (**a**–**c**), respectively. Black line presents data obtained after fitting of Curie’s law.

Figure 10 shows the characteristics of the inverse of magnetic susceptibility depending on temperature along with an extrapolating line for all gadolinium-doped materials. A negative intersection point of the fitting curve with the temperature axe indicates the antiferromagnetic nature of magnetic interactions in tested materials. The presence of antiferromagnetic interactions in the samples discussed results in reduced ferromagnetic order, which is manifested by the occurrence of weak ferromagnetism and quite low magnetization values.

## 4. Conclusions

The tested ceramic materials with an Aurivillius-type structure were synthesized by solid-phase synthesis from simple oxides. The synthesis resulted in single-phase materials characterized by a perovskite-like structure, which is typical for Aurivillius-type structures. XRD analysis confirmed that BGFT-x ceramics are single-phase with an orthorhombic *Fm*2*m* structure. SEM images revealed that doping with Gd^3+^ ions alters the microstructure, changing the grain edges to be rounded. EDS analysis verified the chemical composition and confirmed the absence of impurities. Magnetic testing revealed that the BFT ceramic material exhibits ferromagnetic properties at low temperatures (*T* = 10 K and *T* = 77 K) but becomes paramagnetic at room temperature. Notably, doping BFT with Gd^3+^ ions significantly enhances its magnetic properties. The doped Aurivillius-type materials (BGFT2, BGFT4, and BGFT6) exhibit ferromagnetic properties even at room temperature. This improvement in magnetic properties at higher temperatures is crucial for potential applications and lays the groundwork for further modifications of the chemical composition of Aurivillius-type ceramic materials to achieve even better magnetic properties at elevated temperatures.

## Figures and Tables

**Table 1:** Basic parameters of hysteresis loops measured for all materials doped with Gd^3+^ ions, i.e., for BGFT2, BGFT4, and BGFT6.

$x_{{Gd}^{3+}}$	*T* = 10 K			*T* = 77 K			*T* = 300 K		
	$H_{c}[Oe]$	$H_{ex}[Oe]$	$M_{r}[\frac{emu}{g}]$	$H_{c}\left[Oe\right]$	$H_{ex}\left[Oe\right]$	$M_{r}\left[\frac{emu}{g}\right]$	$H_{c}[Oe]$	$H_{ex}[Oe]$	$M_{r}[\frac{emu}{g}]$
0.2	−45.76	−32.96	0.0079	−75.85	−30.94	0.0026	−91.41	−5.71	0.0010
0.4	−78.43	−28.90	0.0186	−241.63	−3.79	0.0097	−315.39	45.55	0.0049
0.6	−78.39	−47.50	0.0275	−256.04	−44.88	0.0127	−385.13	−6.76	0.0067

## Data Availability

The original contributions presented in the study are included in the article, further inquiries can be directed to the corresponding author.
